# Microstructure and Mechanical Properties of a Cold-Rolled Ultrafine-Grained Dual-Phase Steel

**DOI:** 10.3390/ma11081399

**Published:** 2018-08-10

**Authors:** Zhiyi Pan, Bo Gao, Qingquan Lai, Xuefei Chen, Yang Cao, Manping Liu, Hao Zhou

**Affiliations:** 1 Nano and Heterogeneous Materials Center, School of Materials Science and Engineering, Nanjing University of Science and Technology, Nanjing 210094, China; zhiyipan0602@163.com (Z.P.); gaobo@njust.edu.cn (B.G.); y.cao@njust.edu.cn (Y.C.); 2 Herbert Gleiter Institute of Nanoscience, Nanjing University of Science and Technology, Nanjing 210094, China; 3 State Key Laboratory of Advanced Special Steel, Shanghai University, Shanghai 200444, China; 4 State Key Laboratory of Nonlinear Mechanics, Institute of Mechanics, Chinese Academy of Sciences, Beijing 100190, China; chenxuefei@lnm.imech.ac.cn; 5 School of Engineering Science, University of Chinese Academy of Sciences, Beijing 100049, China; 6 School of Materials Science & Engineering, Jiangsu University, Zhenjiang 212013, China; manpingliu@ujs.edu.cn

**Keywords:** dual phase steel, cold-rolling, intercritical annealing, microstructure refinement

## Abstract

A new processing route to produce Ultrafine-Grained Dual-Phase steel has been proposed, involving cold-rolling and subsequent intercritical annealing of a fibrous ferrite–martensite starting structure. Ultrafine-grained DP (UFG-DP) steel with an average ferrite grain size of about ~2.7 μm and an average martensite island size of ~2.9 μm was achieved. Tensile testing revealed superior mechanical properties (the ultimate tensile strength of 1267 MPa and uniform elongation of 8.2%) for the new DP steel in comparison with the fibrous DP steels. The superior mechanical properties are attributed to the influence of microstructure refinement on the work-hardening and fracture behavior.

## 1. Introduction

The increasing demand for weight reduction with high safety performance in the automotive industry has led to the continuous development of advanced high strength steels (AHSS), such as dual-phase (DP) and transformation-induced plasticity (TRIP) steels [1,2,3,4]. Compared to other high-strength steel grades, DP steels involve a leaner alloy addition and can be produced using well-adapted processes, while they show attractive balance between strength and formability [5,6,7,8]. However, the new wave of fuel-economy regulations demands better combination between strength and ductility/toughness, so that the stringent design requirements can be met. One of the promising approaches achieving this desire is to produce DP steels with ultrafine grain size. It has been well known that grain refinement of DP steels leads to huge strength enhancement without apparent sacrifice of plasticity [9,10,11,12,13,14,15,16,17,18,19,20]. While, single-phase UFG materials usually show high strength but limited ductility [19]. Previous studies [16] attributed the benefits of UFG-DP to optimizations of strain hardening rate, martensite plasticity and interface cohesion. A better understanding of the grain refinement is worthy to be studied further to optimize mechanical properties of UFG-DP steels. 

However, the successful production of UFG-DP remains a challenge, which relies on starting with the fine-scale microstructure and maintaining the structural scale during intercritical annealing. The fine-scale initial microstructures can be achieved by severe plastic deformation (SPD). Shin et al. [21] and Park et al. [22] applied the equal channel angular pressing (ECAP) (accumulated strain of ~4) to generate a ferritic initial structure with the grain size of ~1 μm and ~0.2 μm, respectively. Tsuji [23] used the technique of accumulative roll bonding (ARB) to refine the initial ferrite grain size to 1.3μm with the equivalent strain of 4.8. In addition, fast heating during intercritical annealing was found to be important to maintain the ultrafine grain size, which is allowed by the pinning of ferrite grain boundaries by the formation of austenite islands [6,7,24,25,26]. It is reported that the grain growth of ferrite can only be significantly suppressed with the heating rate of 300 K/s [17]. Moreover, playing with the interaction between ferrite recrystallization and austenite formation, the production of UFG-DP microstructures can be eased by increasing the Manganese content, which decreases the temperature of austenite formation and more efficient suppression of ferrite grain growth was resulted in [6].

The abovementioned techniques involving SPD, fast heating and increased Manganese alloying are, on the other hand, bringing barriers to the industrial manufacturing of UFG-DP. The objective of the present work is to propose a new processing route for UFG-DP by designing the initial microstructure before intercritical annealing. The initial microstructure is a cold-rolled fibrous ferrite–martensite dual-phase steel. In this fine-scale initial structure, the dispersion of martensite plates/islands offers high density of preferred nucleation sites for austenite formation, which enhances the efficiency of pinning the migration of ferrite grain boundaries during intercritical annealing. This processing route proved effective in achieving UFG-DP microstructures. The mechanical properties of the UFG-DP were examined by uniaxial tensile tests. The influence of microstructure refinement on the work-hardening and fracture behavior are discussed.

## 2. Materials and Methods 

The chemical composition of the steel was shown in Table 1, which was determined using the vacuum emission spectroscopy. The ingot was prepared in a 20 kg vacuum induction furnace with a thickness of 50 mm. The slabs were cut to samples of 40 × 30 × 8 mm for processing and mechanical testing. 

For the selection of processing temperatures, A1 and A3 temperatures were calculated using Equations (1) and (2) [27]:

(1) A1=723−10.7Mn−16.9Ni+29.1Si+16.9Cr+290As+6.38W

(2) $$A3=910-203C^{0.5}-15.2\mathrm{Ni}+44.7\mathrm{Si}+31.5\mathrm{Mo}+104V+13.1W$$

**Figure 1:**
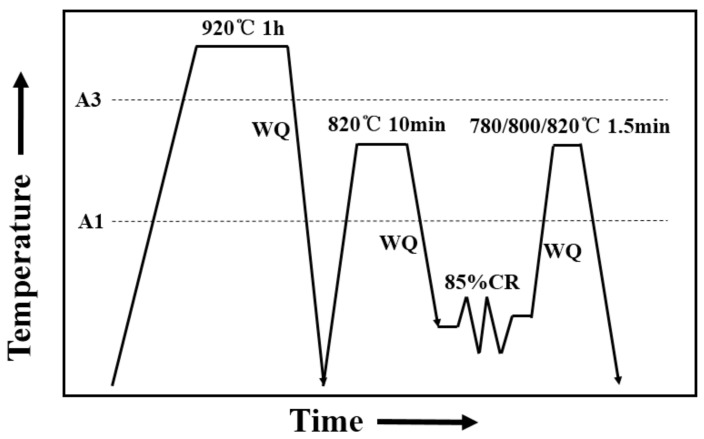
Thermomechanical cycle used to produce UFG-DP steels.A1 and A3: austenite formation’s start and finish temperatures during heating respectively; WQ: water quench; CR: cold rolling.

A1 and A3 temperatures were estimated as 755 °C and 887 °C, respectively. The processing route to develop UFG-DP microstructures is shown in Figure 1. Firstly, the samples were austenitized at 920 °C for 60 min followed by water quench to obtain a martensitic microstructure. Then, an intercritical annealing (820 °C, 10 min) was performed to generate a fibrous ferrite–martensite microstructure. The steel sheet were then cold rolled by 85% reduction in thickness in order to refine the structural scale. Finally, another intercritical annealing was performed on the cold-rolled sheets at 780 °C, 800 °C or 820 °C for 1.5 min, followed by water quenching. During the final intercritical annealing processing, in order to achieve relatively fast heating rate, the steel samples were submitted to the furnace at the temperature of 780 °C, 800 °C or 820 °C. The samples are placed in direct contact with the heated iron blocks for an efficient heat transfer.

Microstructural characterization was carried out along the plane perpendicular to the transverse direction (TD) and parallel to rolling direction (RD). The sectioned specimens were mounted, ground, polished and etched with 5% Nital solution. The microstructures were examined by optical microscope (OM) and scanning electronic microscopy (SEM, Quant 250 FEG). The SEM micrographs were quantitatively analyzed by image analysis following the procedure in [6] so that the volume fraction of martensite and the average grain size of ferrite can be measured.

The steel sheets were cut into dog-bone shaped specimens for tensile testing, with the longititudinal direction parallel to the rolling direction. The gage length, width and thickness of the tensile specimens were 10 mm, 2.5 mm and 1.2 mm, respectively. Uniaxial tensile tests were performed on an electromechanical universal testing machine (LFM-20kN) with a strain rate of 3 × 10^−3^ s^−1^ at room temperature. Following the procedure in the literature [28], load-deformation curves of the samples was tested and in order to get true strain some data processing was done. The displacement of the sample holder was measurement, and the strain was calculated by dividing the gauge length by such displacement, after calibrating the stiffness of the testing machine. The yield strength was defined as the flow stress at 0.2% plastic strain. The uniform elongation was defined by the true strain at the moment of necking according to the Considère ecriterion [29]. Three samples were tested for each microstructural state. The fracture surfaces of the broken tensile specimens were observed under the SEM.

## 3. Results and Discussion

### 3.1. Microstructure Evolution During Processing

**Figure 2:**
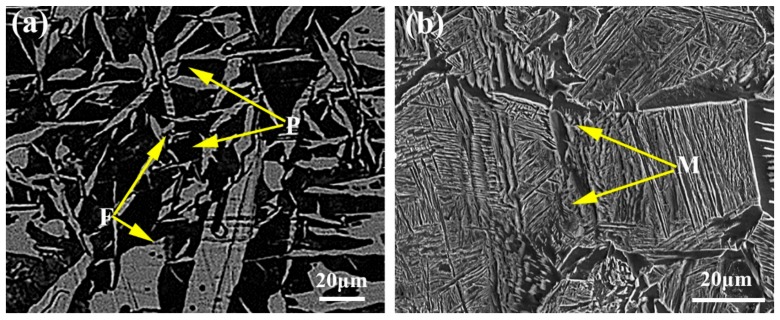
Optical micrographs of (**a**) as-received microstructure; and SEM micrographs of (**b**) the microstructure after annealing at 920 °C for 60 min.

The as-received microstructure consists of a mixture of pearlite and ferrite (Figure 2a). The volume fraction of pearlite was measured as 40%, and the ferrite grain size is 30 μm. After annealing at 920 °C for 60 min, a martensitic microstructure was attained (Figure 2b). The small amount of ferrite is inevitable in this steel grade due to insufficient quenchability.

**Figure 3:**
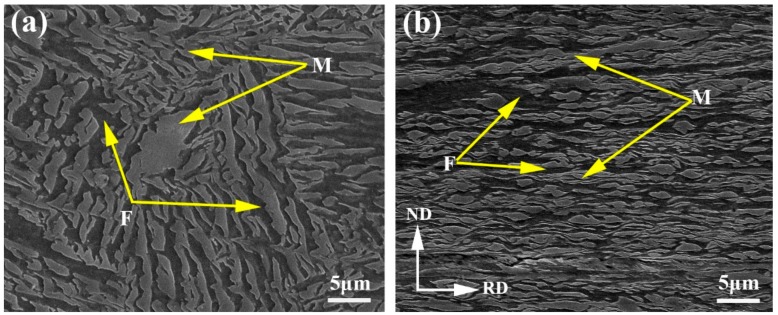
SEM morphologies of the fibrous DP microstructures after (**a**) water quench; and (**b**) cold rolling.

The martensitic initial structure was annealed at 820 °C for 10 min to produce the fibrous DP steels. The microstructure consists of 47 vol % ferrite and 53 vol % martensite (Figure 3a), and the fibrous morphology is considered as inheriting from the lath martensite during intercritical annealing [30]. The distribution of martensite islands is uniform within the microstructure, and banding structure, which is usually significant in Mn-alloyed steels due to Mn segregation, cannot be observed. In order to refine the structural scale, cold-rolling to 85% thickness reduction was performed. As shown in Figure 3b, the dual-phase microstructure is well orientated along the rolling direction, with the mean spacing between martensite islands dramatically reduced. The martensite islands show the features of co-deformation with ferrite during cold-rolling, although the martensitic strength is much higher than ferrite.

**Figure 4:**
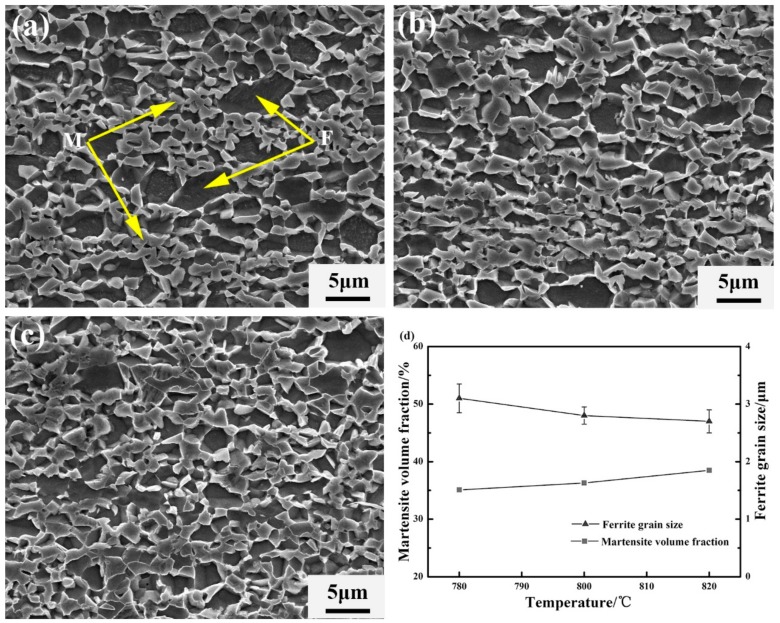
SEM micrographs of deformed ferrite–martensite after annealing for 1.5 min at (**a**) 780 °C; (**b**) 800 °C; (**c**) 820 °C; and (**d**) martensite volume fraction and ferrite grain size.

**Figure 5:**
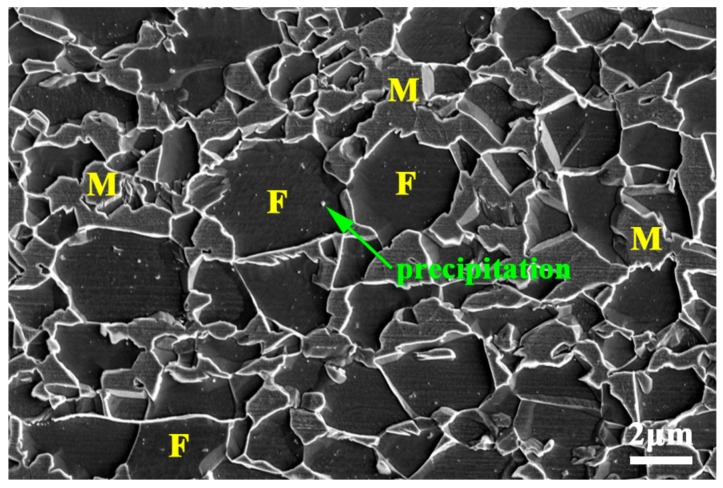
High-magnification SEM micrograph of UFG-DP steels.

**Figure 6:**
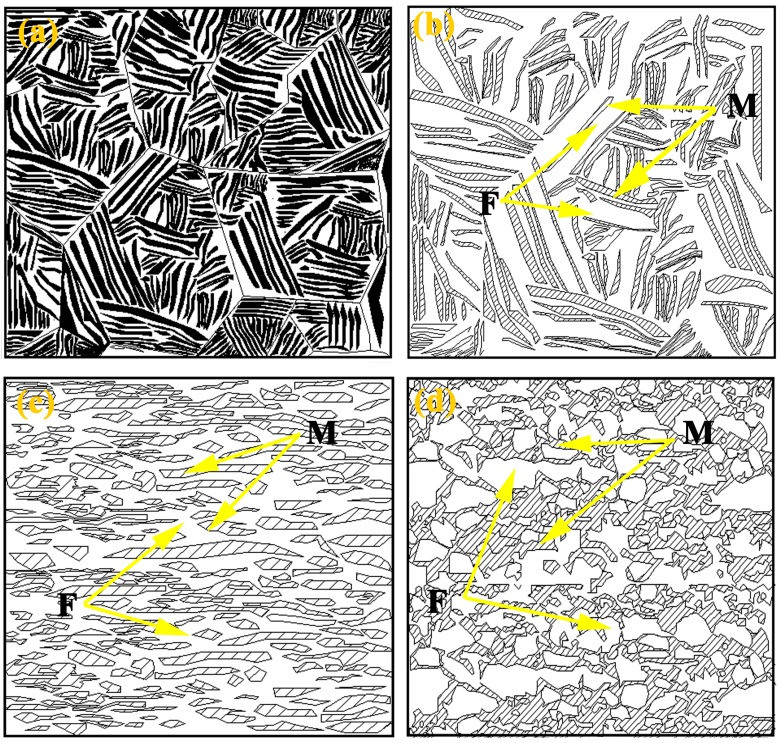
Schematic representation of prevailing microstructure evalution during processing. (**a**) martensitic microstructures; (**b**) fibrous DP microstructures; (**c**) cold rolled fibrous DP microstructures; and (**d**) UFG DP microstructures.

Figure 4a–c shows the microstructures after the final intercritical annealing at 780 °C, 800 °C and 820 °C for 1.5 min, respectively. These microstructures show a uniform distribution of the equiaxed UFG ferrite grains (2~3 μm) with the embedded UFG martensite islands (2~3 μm), indicating that the processing route works well in achieving ultrafine-grained dual-phase microstructures. Compared to the fibrous dual phase microstructure, UFG-DP (annealing at the same temperature 820 °C) microstructure has a different martensite volume fraction, consisting of 38 vol % martensite (Figure 4d). This is mainly attributed to the soaking time. During the processing to obtain UFG-DP, the short soaking time was used in order to avoid substantial grain growth during annealing. However, the short soaking time of 1.5 min probably led to incomplete formation of the austenite. The fibrous DP and the UFG-DP have different martensite volume fractions. Figure 4d also shows that increasing the annealing temperature did not result in a larger ferrite grain size. During intercritically annealing the microstructure as shown in Figure 3b, the austenite phase preferably forms from the existing carbon-rich martensite islands. The amount of austenite phase is increased at a higher annealing temperature, which is indicated by the evolution of martensite volume fraction after quenching. On the other hand, the driving force to recrystallization and grain growth of ferrite is large due to the heavy cold-rolling. The dispersion of easily formed austenite dispersions acts as efficient pinning points to the migration of ferrite grain boundaries, which results in the fact that the fine scale of the structure survived after the heat treatment. However, the recrystallization of ferrite is not fully suppressed, which explains the equiaxed ferrite grain shape. Moreover, as shown in the Figure 5, low-density of precipitations are observed in some ferrite grains. This could be explained by the procedure of the heat treatment. The intercritical annealing was performed on the ferrite–martensite mixture, where the Carbon is mainly in solute and concentrated in the martensite phase. The relatively fast heating rate also helped in restraining carbide precipitation and spheroidization. The contribution of such low-density precipitations to the flow stress is considered to be the second-order effect, when comparing with the effect of grain refinement. The abovementioned microstructure evolution during the processing steps was shown schematically in Figure 6.

### 3.2. Tensile Properties

**Figure 7:**
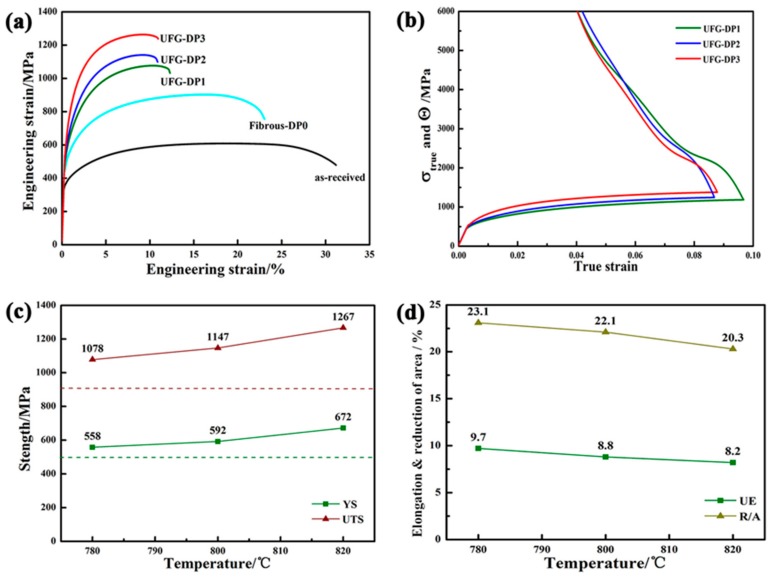
(**a**) Uniaxial tensile engineering strain–stress curves; (**b**) True stress (σ_true_) and strain hardening (Θ) curves from tensile tests. UFG-DP1, 2 and 3 respectively represent the samples after the final intercritical annealing at 780, 800 and 820 °C for 1.5 min; (**c**) Yield strength (YS) and ultimate tensile strength (UTS) at different intercritical temperature. The two dashed lines represent the YS and the UTS of the fibrous DP steels respectively; (**d**) Uniform elongation (UE) and reduction of area (R/A) after annealing at different intercritical temperature.

The engineering stress-strain curves of as-received sample, the fibrous DP steels and the UFG-DP steels are compared in Figure 7a. All the samples show continuous yielding behavior, and the level of flow stress is significantly increased when the microstructure is refined. Figure 7b shows the true stress-strain curves and the corresponding evolution of strain hardening rate of the UFG-DP steels. All the UFG-DP steels show a high strain hardening rate in the initial stages of the deformation, which decreases gradually with strain. This salient strain hardening behavior enables the DP steels to possess a low yield ratio, high tensile strength and relatively high uniform elongation. As compared to the fibrous DP steels, the UFG-DP steels present both higher yield strength and tensile strength (Figure 7c). This could be attributed to the reduced ferrite grain size in UFG-DP. The ferrite in fibrous DP and in UFG-DP could behave differently. In a certain sense, the structural scale of ferrite in fibrous DP is also in UFG regime in that the thickness of the ferrite lamella is about 2 μm. However, the free path of dislocations along the length of the ferrite lamella could be rather large (tens of microns, comparable with the prior austenite grain size), resulting in a large effective ferrite grain size. Indeed, the yield strength of fibrous DP steel is lower than the UFG-DP steels. In addition, although the size of marteniste islands is also reduced in UFG-DP, this is presumably not contributing to the increase in martensitic strength because the main metallurgical factor determining the strength of martensite is carbon content [31].

Moreover, according to Figure 7d, the uniform elongation (UE) of the UFG-DP1, 2 and 3 steel (samples after the final intercritical annealing at 780, 800 and 820 °C for 1.5 min.) is decreased with increasing annealing temperature. When the annealing temperature is increased, the martensite volume fraction (*V*m) is increased while the ferrite grain size (d_f_) is reduced (Figure 4d). Both of these factors tend to decrease the uniform elongation of the DP steels [6,20].

An interesting point of UFG-DP is the excellent uniform elongation when comparing to other UFG single-phase ferrous alloys. It is reported that the ferritic steels with a grain size less than 1μm usually exhibit poor uniform elongation [19], but recent studies on grain refinement in DP steels show that the improvement of strength is not necessarily accompanied by a huge decrease of uniform elongation [6,32]. This could be attributed to the unique effect of simultaneously decreasing the size of ferrite grains and martensite particles. During formation, the plastic strain incompatibility between ferrite and martensite induces a large number of geometrically necessary dislocations (GNDs) in the ferrite areas near the interfaces. The GND sources are increased with larger of the ferrite/martensite interfacial regions [19]. Although the plastic incompatibility between the ferrite and martensite is weakened by grain refinement, the huge dislocation multiplication in ferrite grains and the consequential additional back stress in the local region enables the UFG-DP microstructure to strain harden, resulting in better ductility than UFG single-phase metals [33]. 

### 3.3. Fractography

**Figure 8:**
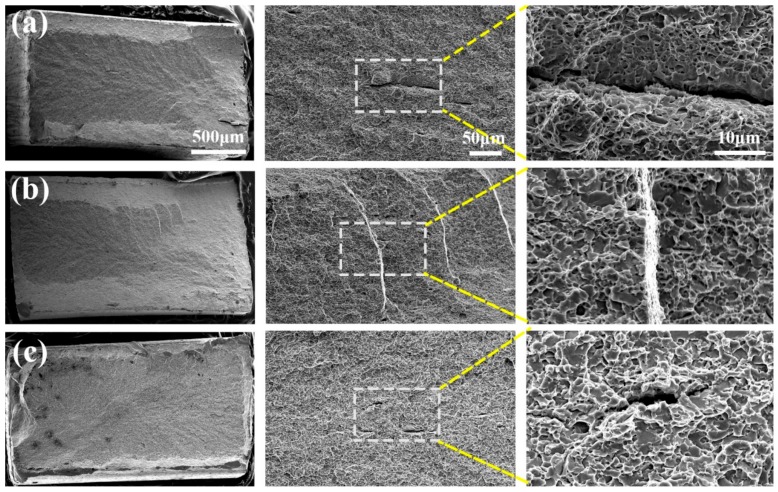
SEM micrographs of the fracture surface of (**a**) UFG-DP1; (**b**) UFG-DP2; and (**c**) UFG-DP3.

Observations of fracture surfaces of the UFG-DP are shown in Figure 8. All fracture surfaces reveal a mixture of cleavage facets and dimples. No large dimples were observed, which indicates that void growth is limited during the fracture processes. Instead, the dimples were all small and shallow. When the intercritical temperature increases, the area fraction of cleavage facets increases and the sample exhibits a more brittle manner. It is reported that the DP steels with a high volume fraction of the martensite could undergo brittle fracture at ambient temperature [6]. The brittle fracture of DP steels is usually induced by a micromorphology of interconnected martensite network [34,35]. Compared to isolated martensite islands, the martensite network in DP steels restricts the flow of plasticity in the ferrite by confining the slip systems [34] and induces a high degree of stress triaxiality. Presumably, the damage nucleation occurs at the martensite phase that has lower toughness and bears higher stress level. Once the martensite fractures, the initiated crack in martensite imposes a concentrated stress field and also a high degree of stress triaxiality in the neighboring ferrite grain. In addition, significant elastic energy, which is stored due to the deformation of high-strength materials, can be released once the crack starts propagating, resulting in unstable crack growth. The abovementioned stress concentration, stress triaxialty and rapid release of elastic energy trigger the cleavage cracking in ferrite neighboring the damaged martensite particles. It is the premature cracking in martensite that controls the fracture resistance and/or fracture strain in the UFG-DP steels. Hence, with an increasing amount of martensite phase, there will be more brittle features on the fracture surface and a lower fracture strain is seen in as shown by the area reduction in Figure 7d. 

## 4. Conclusions

1. Ultrafine-grained ferrite–martensite dual-phase steels were produced by intercritically annealing the cold-rolled fibrous DP microstructure. The generated dual-phase steels consisted of UFG ferrite and uniformly distributed martensite islands. Ferrite grain size and martensite island size were ~2.7 μm and ~2.9 μm, respectively.
2. The UFG-DP steels exhibit an excellent strain hardening behavior, which is affected by the grain refinement of both ferrite and martensite, enabling the simultaneous enhancement of UTS and UE to 1.2 GPa and 8%, respectively.
3. The UFG-DP steels fractured in a partially brittle manner. A mixture of small dimples and cleavage facets was observed in the fracture surfaces. The fracture strain characterized by area reduction was decreased when the volume fraction of martensite was increased.

## Figures and Tables

**Table 1:** Chemical composition (wt %) of the steel.

Element	Content
C	0.19
Mn	1.01
Si	1.46
Fe	Balance
